# Clinical outcomes of prenatal diagnosis of the fetal micrognathia: A case report

**DOI:** 10.1097/MD.0000000000018648

**Published:** 2020-01-24

**Authors:** Jin-Wen Lu, Dan Lu, Xiao-Li Zhang, Jiao Bai

**Affiliations:** Department of Ultrasound, Zhongnan Hospital of Wuhan University, Wuhan, China.

**Keywords:** cleft palate, micrognathia, prenatal diagnosis

## Abstract

**Rationale:**

Micrognathia is a subtle facial malformation characterized by a small mandible and receding chin. Fetal micrognathia is often associated with chromosomal abnormalities, skeletal dysplasia, and various syndromes. Once it is dignosised, detailed fetal malformation screening and chromosome examination should be carried out.

**Patient concern:**

One pregnant woman with suspicion of fetal micrognathia was referred from her local hospital to our hospital for detailed fetal malformation screening and fetal echocardiography. Examination of the fetus was performed using a two-dimensional and three-dimensional ultrasound probe in multiple planes. The fetus showed micrognathia without glossoptosis with features of the inferior facial angle (IFA) ≤50° and his tongue reached anterior mandibular border box during normal movement.

**Diagnoses:**

The fetus was diagnosed as isolated micrognathia prenatally without multisystem abnormalities.

**Interventions:**

Amniocentesis was performed and the fetus was found to carry 46XN with 6q14.1 duplication, the significance of which was unclear.

**Outcomes:**

The fetus was labored through vagina at 38 weeks gestation. A small soft cleft palate was diagnosed after delivery.

**Lessons:**

This case suggests that once prenatal diagnosis of the fetal micrognathia has been made, we should carefully examine the presence of fetus's multisystem developmental abnormalities and due consideration should be given for associated soft cleft palate.

## 1 Introduction

Micrognathia is a subtle facial malformation characterized by a small mandible and receding chin. Fetal micrognathia is often associated with chromosomal abnormalities, skeletal dysplasia, and various syndromes.^[1,2]^ Nowadays, micrognathia can be easily diagnosed via ultrasonography because of its typical ultrasonographic features. During to its association with additional structural or chromosomal abnormalities, detailed fetal malformation screening and chromosome examination should be carried out including cleft palate screening for every fetus.^[3]^ In this case, although we had a detailed prenatal examination of the fetal palate, we still missed a diagnosis of small soft cleft palate. Therefore, the purpose of this case report is to describe the specific ultrasonographic features of the fetus and to remind us that the awareness and scanning skills required to diagnose micrognathia combined with cleft palate is essential for health professionals.

## 2 Case presentation

**Figure 1:**
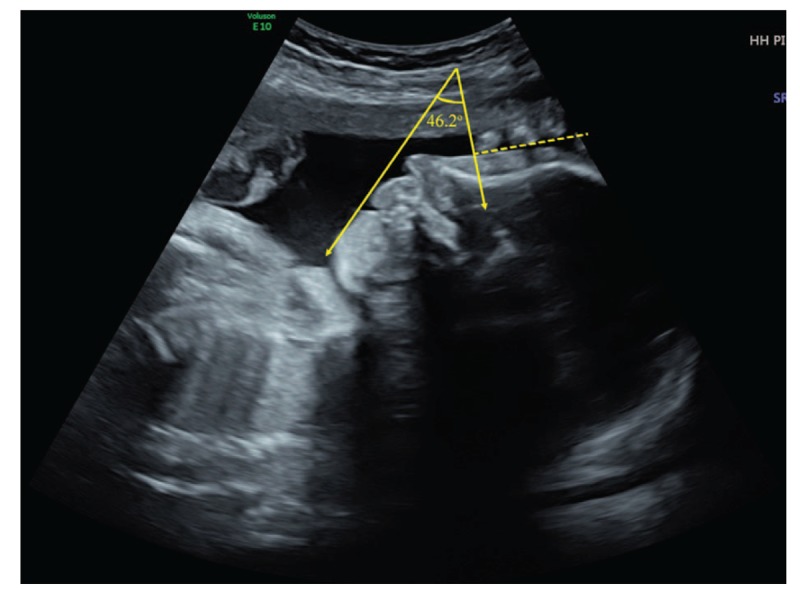
Two-dimensional ultrasound of the fetal facial profile with the IFA demonstrated. IFA = the inferior facial angle.

**Figure 2:**
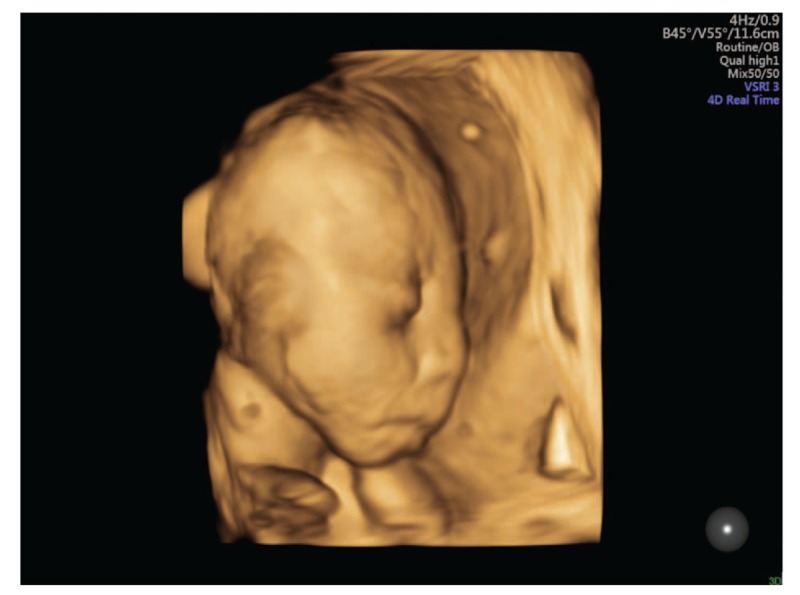
Three-dimensional ultrasound of the fetal facial profile with micrognathia.

**Figure 3:**
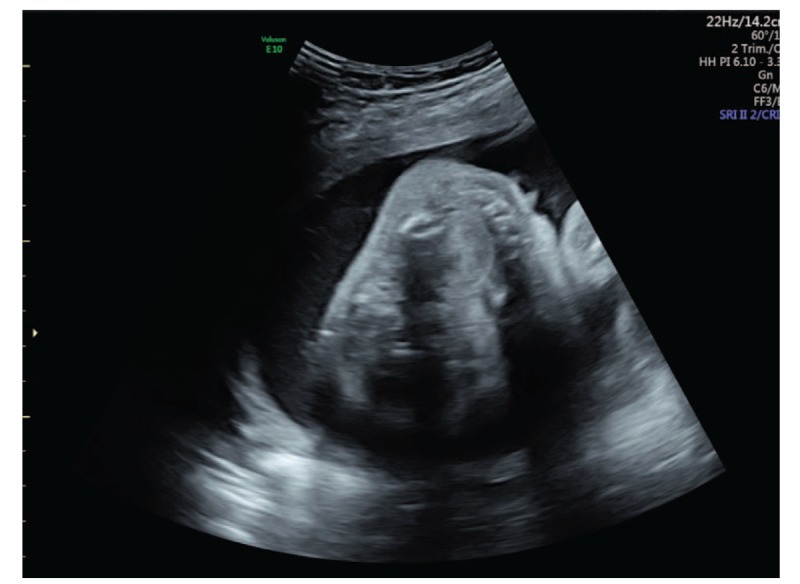
Two-dimensional ultrasound of the fetal tongue reached the anterior mandibular alveolar ridge.

**Figure 4:**
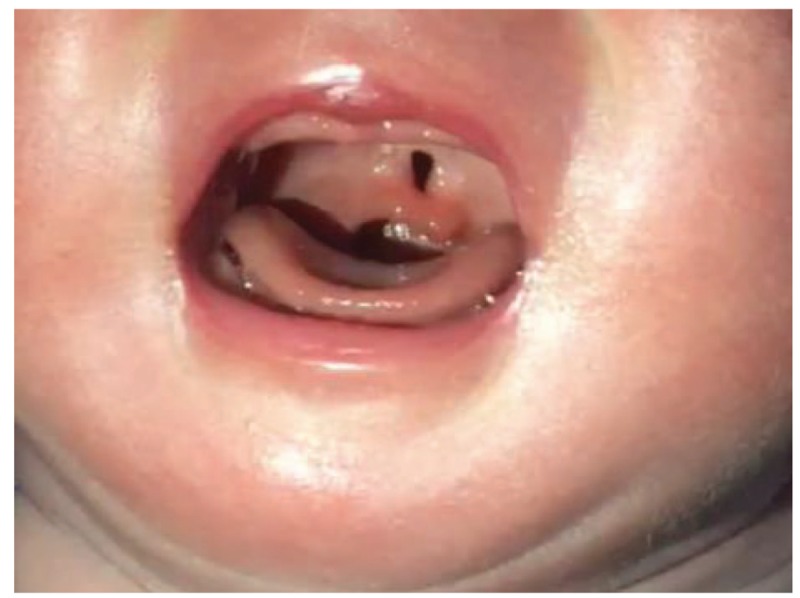
v-shaped soft cleft palate of the neonate.

This is a case report of a 33-year-old Chinese Han woman, gravida 3 para 1. The patient reported low fever that went untreated at about 50 days of gestation. She was not knowingly exposed to teratogens prior to or during pregnancy and did not have a family history of congenital disease. At week 24, the patient received routine examination with abdominal three dimensional ultrasound at local hospital and the result indicated fetal micrognathia. The patient came to our hospital for re-examination at week 26. Conventional sonography revealed the fetus had an the inferior facial angle (IFA) of 46.2° below the lower limit of normal value 50°. The IFA is calculated by measuring the angle made by the cross-section of a line orthogonal to the forehead at the level of the nasofrontal suture and a line from the tip of the mentum to the anterior border of the more protrusive lip on a sagittal view (Fig. 1). Three-dimensional ultrasound of the fetal facial profile also showed micrognathia (Fig. 2). It is known that isolated micrognathia should differentiate with syndromed micrognathia such as Stickler syndrome, 22q11.2 deletion syndrome, and Pierre Robin sequence. So we evaluated for glossoptosis by viewing the fetus in profile to watch the echogenic tongue for 20 to 30 minutes to see if it was either posteriorly displaced or reached the anterior mandibular alveolar ridge during normal movements. The tongue in the fetus reached the anterior mandibular alveolar ridge (Fig. 3) so we excluded glossoptosis. The rest of multiple-system ultrasound characteristics were normal except the polyhydramnios. Considering the patient's demand for fertility, chromosomal abnormalities could not be excluded. The patient underwent puncture and biopsy of the amniotic cavity for karyotype analysis and copy number variants (CNVs), which was 46XN with 6q14.1 duplication that was not related to clear pathogenic information according to the public database resources. These findings suggested a diagnosis of isolated micrognathia. The family decided to continue the pregnancy after consulting obstetricians and pediatricians due to the strong demand for fertility. Routine prenatal monitoring were made until the fetus was labored through vagina at 38 weeks gestation. The neonate weighed 3000 g and the body was 50 cm long with Apgar scores of 9 and 10 at 1 and 5 minutes, respectively. A physical examination revealed micrognathia without glossoptosis and post-delivery airway intervention. The neonate also exhibited a narrow V shaped soft cleft palate without cleft lip but no obviously feeding difficulties (Fig. 4). The neonate's parents then consulted surgeons of stomatology hospital and received the suggestion that the newborn can undergo surgery after 8 months.

## 3 Discussion

Micrognathia is typically diagnosed without definitive metrics. Numerous studies using more standardized objective methods have been performed including measuring fetal jaw size and micrognathia, mandibular and maxillary widths ratio, the fronto-naso-mental (FNM) angle and the IFA.^[3,4–8]^ Matthew et al^[9]^ compared all these methods and found that IFA was both easier and time-saving to measure accurately than other methods. Merrifield introduced the IFA was calculated by measuring the angle made by the cross-section of a line orthogonal to the forehead at the level of the nasofrontal suture and a line from the tip of the mentum to the anterior border of the more protrusive lip on a sagittal view.^[10]^ Micrognathic fetuses were defined as having IFA values <50°.^[8]^ In this case, the IFA values of the fetus was 46.2° and hence micrognathia was diagnosed. Micrognathia is often associated with chromosomal abnormalities such as trisomies 13 and 18, various malformations such as skeletal dysplasia and facial clefts, and various syndromes for instance Stickler syndrome, 22q11.2 deletion syndrome, and Pierre Robin sequence. Vettraino et al^[11]^ made a retrospective review of fetuses and infants with the prenatal diagnosis of isolated micrognathia for April 1990 to August 2001. The results showed that 15 fetuses had isolated micrognathia by prenatal sonogram. After neonatal examination, 14 of 15 were found to have at least 1 additional abnormality. Eleven had a cleft of the soft and/or hard palate. Seven (54%) of 13 live-born neonates had mild to severe airway obstruction that required intervention. Four (31%) of 13 existed feeding difficulties of varying duration. They concluded that if micrognathia is the only sonographic finding identified, physicians and families should be prepared for a possible respiratory difficulty at delivery, the presence of a cleft palate, and/or developmental delay. Therefore, once fetal mandible malformation is found, attention should be paid to perform detailed ultrasound examination for other organs in case of misdiagnosis. In this case, we carefully examined multiple systems of the fetus. There was no skeletal system malformation, no syndrome of primary mandibular dysplasia. The chromosome examination also excluded pathogenic abnormalities. Although we presented a detailed prenatal examination for the fetal palate, we still missed the diagnosis of small soft cleft palate. On the basis of the published data, Price et al^[12]^ suggested that the micrognathia does induce the cleft palate in human and animal. It is known that the fetal palate includes the hard palate in front and soft palate behind. Hard palate runs behind and horizontally of the incisive foramen, and soft palate or velum curves downwards and backwards from the posterior part of the hard palate and ends in the uvula. Owing to the failure of the fetal palate's fusion, the cleft usually happens in midline. It is commonly associated with clefts of the lip and alveolus, but isolated clefts of the secondary palate account for 25% to 80%.^[13]^ Visualization of soft palate is difficult because of maxillary shadow. He et al^[14]^ reported 5 routine sections to examine the fetal lip and palate thoroughly: coronal section of nose and lip showing the nose and upper lip, horizontal section of upper jaw showing alveolar ridge, midline sagittal section showing secondary palate's fusion line, obliquely hard palate coronal section of oral fissure showing hard palate, and obliquely soft palate coronal section of oral fissure showing soft palate. Thus, in this case, possible explanations for failure of detection of cleft palate include: satisfied images are difficult to obtain through limited scan angle due to the micrognathia and the small mouth crack, or mistaking the tongue filled in soft cleft palate for an intact palate due to their similar acoustic impedance. Angled insonation and three-dimensional ultrasonography helps to get better picture of fetal palate. However, the best method to analyze the palate is still under debate on multiplanar or tomographic reconstructions obtained with three-dimensional ultrasonography. Various specific views have been advocated to overcome maxillary shadow, such as the “reverse face” view, the intraoral “enface” view, the “flipped face” view, “angled insonation,” the “axial underside” view, the “surface-rendered oropalatal sonographic” view, and the “oblique face” view. However, none of above has received general agreement.^[15]^ Delayed detection of the cleft palate was more likely in narrow, isolated clefts of the soft palate, though it occurred in all cleft sizes.^[16]^ To analyze the fetal palate, Tutschek et al^[17]^ suggested that there are 2 mandatory rules to obey. Firstly, sonographers should confirm the absence of shadowing in the target region by adjusting the starting plane of the volume acquisition. Secondly, sonographers should scrutinize the volume in multiplanar mode before switching to render modes. They also suggested two practical approaches to acquire a volume without shadowing of the hard palate. One approach is to start volume acquisition with frontal insonation in an axial plane that demonstrates the horizontal plate of the secondary palate, the other is to start volume acquisition in a cranially tilted median plane that demonstrates the shadow of the alveolar ridge above the palate.

## 4 Conclusion

The diagnosis of micrognathia has a crucial impact on both prenatal and postnatal outcomes of affected individuals due to its association with additional abnormalities. Once the diagnosis is confirmed, a detailed and systemic sonographic examination of the fetus and an intensive interdisciplinary counseling of the parents are needed. Increased awareness and scanning skills of cleft palate among health professionals are important to prevent unwarranted anxiety from misdiagnosis.

## Author contributions

**Formal analysis:** Xiaoli Zhang.

**Project administration:** Jiao Bai.

**Resources:** Dan Lu.

**Writing – original draft:** Jinwen Lu.

**Writing – review & editing:** Jiao Bai.

## References

[1] Bromley B Benacerraf BR Fetal micrognathia: associated anomalies and outcome. Ultrasound Med 1994;13:529–33. 10.7863/jum.1994.13.7.529 7933015

[2] Paladini D Fetal micrognathia: almost always an ominous finding. Ultrasound Obstet Gynecol 2010;35:377–84. 20373481 10.1002/uog.7639

[3] Luedders DW Bohlmann MK Germer U Fetal micrognathia: objective assessment and associated anomalies on prenatal sonogram. Prenat Diagn 2011;31:146–51. 21268032 10.1002/pd.2661

[4] Rogers GF Lim AA Mulliken JB Effect of a syndromic diagnosis on mandibular size and sagittal position in Robin sequence. J Oral Maxillofac Surg 2009;67:2323–31. 19837298 10.1016/j.joms.2009.06.010

[5] Paladini D Morra T Teodoro A Objective diagnosis of micrognathia in the fetus: the jaw index. Obstet Gynecol 1999;93:382–6. 10074984 10.1016/s0029-7844(98)00414-1

[6] Rotten D Levaillant JM Martinez H The fetal mandible: a 2D and 3D sonographicapproach to the diagnosis of retrognathia and micrognathia. Ultrasound Obstet Gynecol 2002;19:122–30. 11876802 10.1046/j.0960-7692.2001.00622.x

[7] Palit G Jacquemyn Y Kerremans M An objective measurement to diagnose micrognathia on prenatal ultrasound. Clin Exp Obstet Gynecol 2008;35:121–3. 18581766

[8] Nemec U Nemec SF Brugger PC Normal mandibular growth and diagnosis of micrognathia at prenatal MRI. Prenat Diagn 2015;35:108–16. 25224124 10.1002/pd.4496

[9] Matthew G Christopher L Charles H Prenatal identification of Pierre Robin sequence: a review of the literature and look towards the future. Fetal Diagn Ther 2016;39:81–9. 25967128 10.1159/000380948

[10] Merrifield LL The profile line as an aid in critically evaluating facial esthetics. Am J Orthod 1966;52:804–22. 5223046 10.1016/0002-9416(66)90250-8

[11] Vettraino IM Lee W Bronsteen RA Clinical outcome of fetuese with sonographic diagnosis of isolated micrognathia. Obstet Gynecol 2003;102:801–5. 14551011 10.1016/s0029-7844(03)00672-0

[12] Price KE Haddad Y Fakhouri WD Analysis of the relationship between micrognathia and cleft palate: a systematic review. Cleft Palate Craniofac J 2016;53:e34–44. 25658963 10.1597/14-238

[13] Campbell S Prenatal ultrasound examination of the secondary palate. Ultrasound Obstet Gynecol 2007;29:124–7. 17252523 10.1002/uog.3954

[14] He G Zhang H Yang J The prenatal ultrasonic diagnosis of isolate fetal cleft palate. Chin J Med Ultrasound 2014;11:561–70.

[15] Rotten D Levaillant JM Benouaiche L Visualization of fetal lips and palate using a surface-rendered oropalatal (SROP) view in fetuses with normal palate or orofacial cleft lip with or without cleft palate. Ultrasound Obstet Gynecol 2016;47:244–6. 26180023 10.1002/uog.14946

[16] Habel A Elhadi N Sommerlad B Delayed detection of cleft palate: an audit of newborn examination. Arch Dis Child 2006;91:238–40. 16352626 10.1136/adc.2005.077958 PMC2065953

[17] Tutschek B Blaas HK 3D ultrasound and the fetal palate. Re: Qualitative evaluation of Crystal Vue rendering technology in assessment of fetal lip and palate. Ultrasound Obstet Gynecol 2017;50:274–6. 28782232 10.1002/uog.17539

